# Design and Implementation of a Dashboard for Drug Interactions Mediated by Cytochromes Using a Health Care Data Warehouse in a University Hospital Center: Development Study

**DOI:** 10.2196/57705

**Published:** 2024-11-28

**Authors:** Laura Gosselin, Alexandre Maes, Kevin Eyer, Badisse Dahamna, Flavien Disson, Stefan Darmoni, Julien Wils, Julien Grosjean

**Affiliations:** 1 Department of Digital Health, Rouen University Hospital, 1, rue de Germont, Cour Leschevin, Gate 21, 3rd floor, Rouen, 76031, France, 33 659775063; 2 Department of Pharmacy, Rouen University Hospital, Rouen, France; 3 Department of Pharmacology, Rouen University Hospital, Rouen, France; 4 Laboratoire d'Informatique Médicale et d'Ingénierie des Connaissances en e-Santé, U1142, INSERM, Sorbonne Université, Paris, France; 5 INSERM U1096, Rouen University, Normandie University, Rouen, France

**Keywords:** drug-drug interaction, adverse, interaction, information system, warehouse, warehousing, cytochrome, personalized medicine, dashboard, drugs, pharmacy, pharmacology, pharmacotherapy, pharmaceutic, pharmaceutical, medication, visualization, develop, development, design

## Abstract

**Background:**

The enzymatic system of cytochrome P450 (*CYP450*) is a group of enzymes involved in the metabolism of drugs present in the liver. Literature records instances of underdosing of drugs due to the concurrent administration of another drug that strongly induces the same cytochrome for which the first drug is a substrate and overdosing due to strong inhibition. IT solutions have been proposed to raise awareness among prescribers to mitigate these interactions.

**Objective:**

This study aimed to develop a drug interaction dashboard for Cytochrome-mediated drug interactions (DIDC) using a health care data warehouse to display results that are easily readable and interpretable by clinical experts.

**Methods:**

The initial step involved defining requirements with expert pharmacologists. An existing model of interactions involving the (*CYP450*) was used. A program for the automatic detection of cytochrome-mediated drug interactions (DI) was developed. Finally, the development and visualization of the DIDC were carried out by an IT engineer. An evaluation of the tool was carried out.

**Results:**

The development of the DIDC was successfully completed. It automatically compiled cytochrome-mediated DIs in a comprehensive table and provided a dedicated dashboard for each potential DI. The most frequent interaction involved paracetamol and carbamazepine with *CYP450 3A4* (n=50 patients). The prescription of tacrolimus with *CYP3A5* genotyping pertained to 675 patients. Two experts qualitatively evaluated the tool, resulting in overall satisfaction scores of 6 and 5 out of 7, respectively.

**Conclusions:**

At our hospital, measurements of molecules that could have altered concentrations due to cytochrome-mediated DIs are not systematic. These DIs can lead to serious clinical consequences. The purpose of this DIDC is to provide an overall view and raise awareness among prescribers about the importance of measuring concentrations of specific drugs and metabolites. Ultimately, the tool could lead to an individualized approach and become a prescription support tool if integrated into prescription assistance software.

## Introduction

### Background

The enzymatic system of Cytochrome P450 (*CYP450*) is a group of enzymes involved in the metabolism of drugs present in the liver. *CYP450* functions by oxidizing molecules through various chemical reactions. These reactions give rise to active or inactive metabolites, and sometimes among these, to toxic metabolites [1]. They are sometimes involved in drug interactions (DIs), some of which result from their effects on drug-metabolizing enzymes, leading to reversible or irreversible inhibition, or enzyme induction [2]. DIs involving *CYP450* enzymes arise from the concurrent administration of a substrate metabolized by an enzyme and an inhibitor or inducer that shares the same metabolic pathway [3].

While most metabolized drugs are transformed into inactive metabolites, some are converted into active metabolites, such as prodrugs. For instance, codeine, a prodrug of morphine, is demethylated by oxidation by *CYP450 2D6* to produce morphine [4]. These enzymatic reactions involve numerous processes, including prodrug-to-drug conversion, easy drug excretion, and the generation of reactive metabolites, many of which can cause toxicity [5].

The literature documents cases of underdosing or overdosing of drugs due to the concurrent administration of another drug that strongly inhibits or induces the same cytochrome for which the first drug is a substrate. An example is the concurrent prescription of immunosuppressants for the prevention of transplant rejection with azole antifungals. Fluconazole, an enzyme inhibitor of *CYP450 3A4*, increases blood concentrations of tacrolimus (*CYP450 3A4* substrate) by inhibiting its metabolism. This can lead to tacrolimus overdose and subsequent nephrotoxicity. The approach is to monitor the blood concentrations of the immunosuppressant, assess the patient’s renal function, and adjust the dosage during and after the combination [6]. Conversely, if tacrolimus were prescribed with an enzyme-inducing drug for *CYP450 3A4*, there would be a suboptimal dosage of the immunosuppressant, increasing the risk of graft rejection for the patient.

IT solutions have been proposed to assist prescribers in mitigating enzymatic DIs, such as the DDI Predictor server [7], or the Drug Bank database [8]. Current computer modeling approaches for cytochrome metabolism are based on ligands and structures, using techniques such as quantitative structure-activity relationships, machine learning, and molecular dynamics simulations [9]. These methods are available and established in computer-assisted drug design, useful for predicting toxicity in early stages and reducing high failure rates in preclinical and clinical trials [10].

To our knowledge, there is no drug interaction dashboard (DID) specifically linked to a health care data warehouse (HDW) for cytochrome-mediated DIs. In the literature, there are DIDs for pediatric interactions [11], primary care [12], patients infected with SARS-CoV-2 [13], tracking direct oral anticoagulants [14], and implementing DIs within a decision support tool in electronic health records [15].

Other studies related more closely to our topic in pharmacokinetics include an article on pharmacokinetic and pharmacodynamic interactions of antiepileptics in pediatrics [16], and another aiming to establish a DID to provide individual dose recommendations for tacrolimus when coadministered with voriconazole in adult liver transplant recipients [17].

This work follows a previous study [18] whose objective was to provide a structured data source on interactions involving the *CYP450* enzymatic system. These interactions were integrated into the multilingual multiterminology server HeTOP (Health Terminology/Ontology Portal) [19], to support queries in the clinical data warehouse EDSaN (Entrepôt de Données de Santé Normand) [20].

### Objective

The objective of this study is to develop a dashboard for DIs mediated by cytochromes using an HDW in order to display results that are easily readable and interpretable by clinical experts.

## Methods

### Defining Needs With Experts

The first step was to define the requirements for creating a relevant dashboard for clinical practice. This tool was intended for pharmacologists, with whom 3 meetings were conducted. The first meeting aimed to define their needs related to their practice and research issues. The second meeting involved presenting an initial version of the future tool’s prototype and collecting feedback from the pharmacologists. The third meeting presented the final version of the tool and allowed the pharmacologists to use it.

### Using an Existing Model of Interaction Involving Cytochrome Enzymatic System in HeTOP

HeTOP [18] is a multilingual and multiterminology server that includes major health terminologies and ontologies, offering over 3 million concepts available in multiple languages (55 languages) across 100 terminologies or ontologies [21]. Integrating these interactions into the multilingual multiterminology server HeTOP facilitates querying an HDW in this domain. This enables more precise and easier querying of the HDW by directly using the relationships between drugs (substrate, inducer, and inhibitor) and different cytochromes.

Several sources were selected to feed information into the HeTOP server: the University Hospitals of Geneva (HUG) table regarding cytochrome P450 and P-glycoprotein–mediated DIs [22], Drug Bank [8], data from the Belgian Center for Pharmacotherapeutic Information [23], Drug Interactions Flockhart Table [24], US Food and Drug Administration (FDA) [25], and DDI Predictor [7]. Information was integrated manually or automatically when the source structure allowed for it.

### EDSaN and its Connection With HeTOP

The EDSaN [20] data warehouse was put into production in 2020. To date, it contains data from 2 million patients. This HDW was developed internally at the Rouen University Hospital. EDSaN collects all clinical information from the hospital software systems. The data are structured, such as medication prescriptions, and unstructured, such as medical reports. EDSaN is linked to HeTOP, allowing comprehensive queries. HeTOP is used within EDSaN as a semantic assistant for creating queries (textual or structured data), as well as for an automatic annotator to structure data from medical reports.

### Design and Development of a Program for Automated Detection of Cytochrome-Mediated Drug Interactions

The first step was to create an SQL query on the HeTOP Oracle database. This query aimed to select powerful substrate drugs (by unit of Common dispensing [UCD] codes and drug component labels) and drugs that are either powerful inducers or inhibitors of the same cytochrome (by UCD codes and drug component labels). The resulting table from this query included metadata such as cytochrome type, substrate drug component, relationship type, inducer or inhibitor drug component, substrate UCD, and inducer and inhibitor UCDs.

The second step was to filter all UCD codes present at the Rouen University Hospital using unique UCD selections from EDSaN. A Python program was written in the Jupyter programming environment (Jupyter Notebook). A new table containing relevant UCD codes was exported, with the same columns as before.

Using this table, template queries (eg, UCD1 substrate or UCD2 substrate) and (UCD3 inducer or UCD4 inducer) were automatically generated using a Python program. These queries were used to query EDSaN; finally, another Python program was written to call the EDSaN web service. The output format of this service was in JSON, including metadata such as the number of hospitalization days, prescriptions, and affected patients. These data feed the dashboard. The experimentation period was from June 1, 2021, to September 30, 2021.

In addition, for clinical relevance, another dashboard was created involving the prescription of medication and genotyping during the same hospitalization day, at the request of pharmacologists. This was done and extracted from data between January 1, 2019, and September 30, 2021, to ensure a sufficient number of patients.

**Figure 1.**
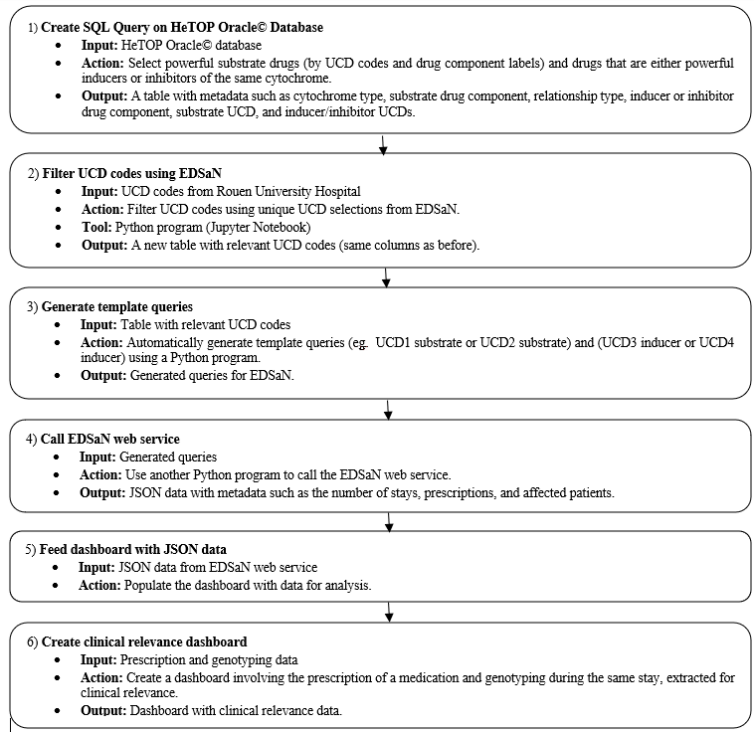
Flowcharts to illustrate data processing steps and the overall architecture of the dashboard. EDSaN: Entrepôt de Données de Santé Normand; HeTOP: Health Terminology/Ontology Portal; UCD: unit of common dispensing.

Figure 1 illustrates the data processing steps and the overall architecture of the dashboard.

### Dashboard Development

A prototype was designed to illustrate the desired visualization and elements to be included in the dashboard. An IT engineer was responsible for developing the technical aspects of the dashboard, using an Agile methodology with iterations between the health care professional in charge of the project (LG) and the IT engineer (KE).

### Visualization

**Figure 2.**
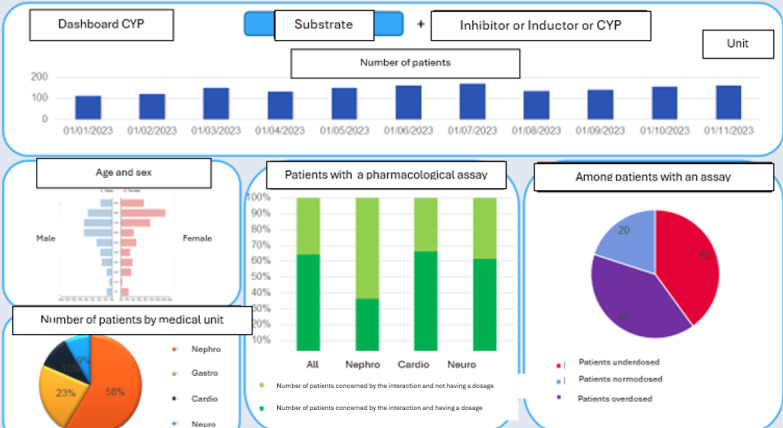
Prototype of the desired visual output.

Figure 2 below depicts the prototype presented to the IT engineer. This prototype was created to guide the programmer in generating the desired visualization. Overall, bar charts and pie charts were chosen to represent the patients affected, as they appeared to be the most suitable for easy and rapid interpretation by clinical experts.

### Qualitative Evaluation of the Final Product

A satisfaction questionnaire was designed for qualitative evaluation (Multimedia Appendix 1). The Computer Usability Satisfaction Questionnaire was used for this purpose [26]. The scores were categorized into several sections to refine the analysis: an overall score, a score for system usefulness, a score for information quality, a score for interface quality, a score for ergonomics, and a score for general satisfaction.

### Ethical Considerations

This study does not include human subjects research (no human subjects experimentation or intervention was conducted) and so does not require institutional review board approval.

## Results

### Overview

The query performed in HeTOP yielded 14,876 lines for analysis. Each line corresponded to the names of drug substances and their corresponding UCD codes. An example line is shown in Table 1 below. Columns 1-4 originate from the knowledge base. Columns 5 and 6 are the result of the automated retrieval of UCD codes for the respective substances. Column 7 represents the final query to interrogate EDSaN. The response time for a query on the interface was between 2 and 5 seconds.

The number of unique UCD codes existing at the Rouen University Hospital was 2156. Some lines (n=49), which had the same molecule as both the substrate and inhibitor or inducer, were removed. Ultimately, there were 14,827 lines of possible interacting substance combinations. Calling the web service for all possible combinations took approximately 7 minutes.

As a single molecule could have multiple corresponding UCD codes, there were initially 4,455,359 possible interaction combinations before processing. Subsequently, the file was filtered with the UCD codes existing at the Rouen University Hospital, resulting in 99,952 possible combinations. These combinations are the result of the “declination” of the UCDs multiplied by the possible pairs of interactions between substances extracted from HeTOP.

**Table 1.** Example of a line from the generated table.

Type of cytochrome	Substrate	Type of relation	Inhibitor or inducer	UCD of substrate	UCD of inhibitor or inducer	Final query
CYP450 CYP1A2	FlecainidFLECAINID	p_inhib	NorfloxacinNORFLOXACIN	3400890352246 or 3400892468143 or 3400892468204 or 3400892468372 or 3400892468433 or 3400892498478 or 3400892635408 or 3400893089811 or 3400893096093 or 3400893104170 or 3400893114117 or 3400893123560 or 3400894250951	3400891057980 or 3400892475356 or 3400892482859 or 3400892555980 or 3400892556062 or 3400892556123 or 3400892558363 or 3400892563336 or 3400892571140 or 3400892686783 or 3400892701325 or 3400892884516	(3400890352246 or 3400892468143 or 3400892468204 or 3400892468372 or 3400892468433 or 3400892498478 or 3400892635408 or 3400893089811 or 3400893096093 or 3400893104170 or 3400893114117 or 3400893123560 or 3400894250951) and (3400891057980 or 3400892475356 or 3400892482859 or 3400892555980 or 3400892556062 or 3400892556123 or 3400892558363 or 3400892563336 or 3400892571140 or 3400892686783 or 3400892701325 or 3400892884516)

### Drug-Drug Interactions

During the period from June 1, 2021, to September 30, 2021, the most frequent interaction involved paracetamol (substrate) and carbamazepine (inducer) with *CYP450 3A4* (n=50 patients). These results are presented in Table 2.

**Table 2.** Results of the 8 most frequent concurrent drug-drug prescriptions in Entrepôt de Données de Santé Normand.

Cytochrome	Substrate	Type	Inhibitor or inducer	Number of patients	Number ofhospitalization days	Number of prescriptions
CYP3A4	Paracetamol	p_induc	Carbamazepine	50	55	55
CYP3A4	Atorvastine	p_inhib	Ticagrelor	25	26	26
CYP3A4	Paracetamol	p_inhib	Verapamil	21	21	21
CYP2B6	Tramadol	p_inhib	Clopidogrel	20	20	20
CYP3A4	Pantoprazole	p_inhib	Ticagrelor	13	14	14
CYP2D6	Oxycodone	p_inhib	Paroxetine	12	12	12
CYP2D6	Ondansetron	p_inhib	Paroxetine	12	12	12
CYP3A4	Paracetamol	p_inhib	Ticagrelor	12	12	12

### Drug-Genotype Interactions

From January 1, 2019, to September 30, 2021, the prescription of tacrolimus with *CYP3A5* genotyping pertained to 675 patients. The prescription of clopidogrel with *CYP2C19* genotyping pertained to 804 patients. These results are presented in Table 3.

**Table 3.** Results of the 2 most frequent concurrent drug-genotype prescriptions in Entrepôt de Données de Santé Normand.

Cytochrome	Substrate	Type	Inhibitor or inducer	Number of patients	Number of hospitalization days	Number of prescriptions
CYP3A5	Tacrolimus	Mutation	Genotype CYP3A5	675	1411	3336
CYP2C19	Clopidogrel	Mutation	Genotype CYP2C19	804	823	828

### Dashboard Views

**Figure 3.**
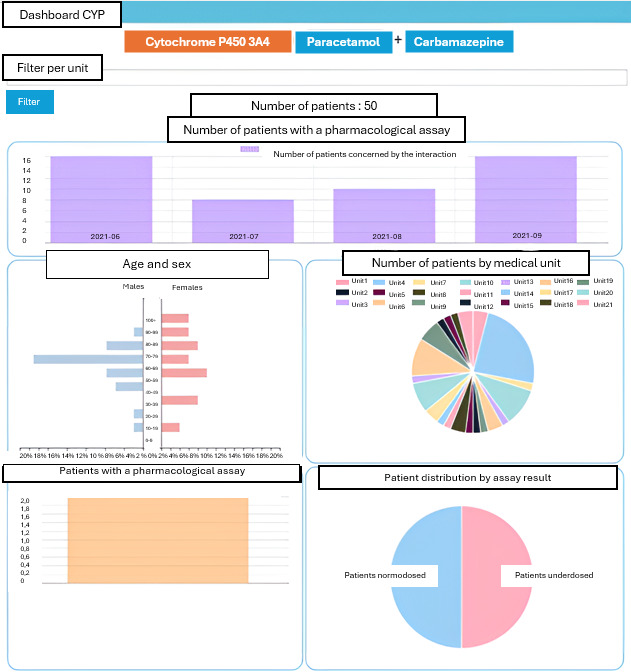
Example of a drug-drug case: paracetamol and carbamazepine.

The dashboards (Figures3  4) are described as follows:

1. The first section provides a temporal view of the number of patients affected by the interaction over time. A filter-by-care service (medical unit) is provided.

2. In the middle of the left side of the dashboard, the distribution by gender and age is displayed.

3. In the middle of the right side of the dashboard, the distribution by medical unit of patients affected by the potential interaction is shown.

4. At the bottom left, the coverage of pharmacology dosage is represented as a percentage.

5. Finally, at the bottom right, the graph illustrates the dosage result by patient status among those who underwent a dosage of the relevant molecule.

**Figure 4.**
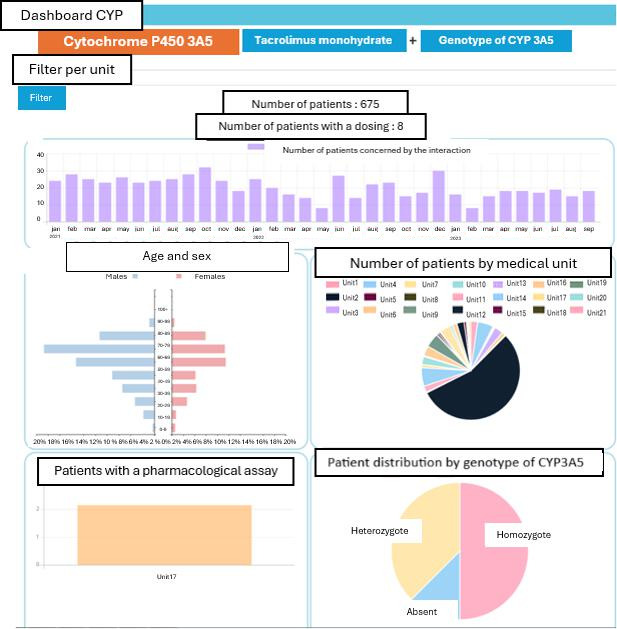
Example of a drug-genotype case: tacrolimus and CYP3A5 genotyping.

### The Computer Usability Satisfaction Questionnaire

**Figure 5.**
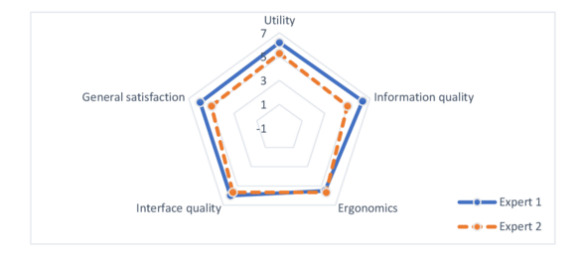
Radar chart representing quality questionnaire results across 5 criteria.

Figure 5 represents the results of the Computer Usability Satisfaction Questionnaire. In total, 2 experts evaluated the dashboard and independently completed the questionnaire. Ratings were given on a scale from 0 (strongly disagree) to 7 (strongly agree). For expert 1 and expert 2, respectively, the system’s usefulness was rated 6.2 and 5.3, information quality was rated 6.3 and 5, usability was rated 6 and 5.7, and overall satisfaction was rated 6 and 5. Remarks for improving the tool included “distinguishing between pharmacogenetic testing and DIs” and “verifying activity levels (number of tests).” In response to the question : “Do you think this tool can be considered a means of communication for raising awareness about necessary pharmacology testing?” the most prominent response was, “Yes, both for performing pharmacogenetic tests and for the need to monitor immunosuppressant concentrations in patients at risk of drug interactions with enzyme inhibitors like azole medications (CYP3A4 inhibition).” In other remarks, the experts noted that it was important to “clearly distinguish drug interactions from pharmacogenetic/drug combinations in the titles.”

## Discussion

### Principal Findings

This dashboard concerning DIs due to cytochromes provides an overall view and visual representation of all the patients affected at the Rouen University Hospital over a given period. This tool will allow pharmacologists to quickly assess these interactions, decide on the relevance of conducting studies based on the patient groups highlighted by the dashboard, or even engage in epidemiological research to sensitize clinicians to prescribe more pharmacological tests for several molecules and have personalized medicine.

It is important to note that these dashboards are dependent on both the knowledge base and feedback from pharmacologists regarding the preselection of interactions to filter and select for clinical relevance.

At the Rouen University Hospital, the testing of molecules that may experience decreased or increased concentrations due to interactions with enzyme inhibitors or inducers is not systematic. The patient’s physiological state can change (such as hepatic insufficiency), or an infection may require the prescription of certain antimicrobials that influence drug metabolism through cytochromes.

These interactions can lead to serious clinical consequences. In cases of therapeutic underdosing of tacrolimus, for instance, there can be graft rejections. In cases of overdosing, adverse drug effects can be exacerbated.

A recent study was conducted by the pharmacology department regarding the prescription of clopidogrel and *CYP450 2C19* genotyping. Clopidogrel is an antiplatelet agent and a prodrug, meaning it needs to undergo hepatic transformation to become an active substance that will be effective. This transformation or metabolism is carried out by the *CYP450 2C19* enzyme. If the enzyme is mutated in the patient, the transformation does not occur properly, and clopidogrel will not be effective. This can lead to serious clinical consequences, such as stroke. Given that this medication is prescribed after an initial stroke, there are risks of recurrence in such cases. If systematic genotyping of *CYP450 2C19* is conducted for each clopidogrel prescription, therapeutic adjustments can be made quickly, such as changing the medication.

In this initial version of the dashboard, only the most frequent interactions have been selected. The most common interaction involves paracetamol (substrate) and carbamazepine (inducer) with *CYP450 3A4* (n=50 patients). In this case, carbamazepine induces the metabolism of paracetamol, resulting in a lower blood concentration than if it were administered alone. This interaction is often overlooked by clinicians as being clinically irrelevant. Frequently, the dosage of paracetamol is increased, or another analgesic is prescribed if the patient is in pain.

Subsequently, the genotyping and DI dashboard highlighted that the prescription of tacrolimus with *CYP3A5* genotyping pertained to 675 patients. Tacrolimus is an immunosuppressive agent used in graft rejection prevention, and it undergoes both enzyme induction and inhibition. For patients on immunosuppressants, certain infections may occur, leading to the prescription of antifungals such as voriconazole. These are known to inhibit *CYP3A4* and *CYP3A5*, resulting in an increase in tacrolimus blood concentration. Clinicians therefore measure tacrolimus levels to adjust the dose during and after anti-infective treatment.

The qualitative evaluation of the dashboard revealed that both experts found it to be useful, providing quality information, user-friendly, having a good interface, and generating overall satisfaction. These criteria can be improved, particularly in terms of the visual presentation differences between the drug-drug interaction and genotyping and DI dashboards.

To the best of our knowledge, this is the first dashboard concerning DIs due to cytochromes. Unlike the other mentioned approaches, this work is more comprehensive and generic, as it encompasses a knowledge base containing a large number of molecules. The downside is that maintaining and updating this knowledge base is essential for the approach to be as exhaustive as possible, which requires significant time.

It is important to note an issue regarding access to testing and genotyping in the EDS (Entrepôt de Données de Santé or Health Data Warehouse). They are not all well-structured, but work is ongoing to improve this. Nevertheless, the infrastructure and methodology in place remain unchanged. In addition, for the genotyping and DI dashboard, the dosage graphs only consider cases where genotyping was done, not cases where the molecule (such as tacrolimus) was dosed.

### Limitations

#### Selection of Relevant Tests

For a given medication, multiple tests can be conducted (at Time+2h, peak concentration, residual concentration, etc). An expert had to manually select the relevant tests to be included in the “patients covered by a test” section of the dashboard. This manual selection process could introduce bias, as it relies on expert judgment, which may vary.

#### Lack of Standardization

The biological tests are not standardized with LOINCs (Logical Observation Identifiers Names & Codes), which makes manual sorting a labor-intensive task. Once the corresponding code is chosen, it must be matched with existing standards. If multiple tests are selected, determining the appropriate standard to apply becomes challenging. Although this issue did not occur with the examples we provided, it is a potential limitation that could arise with different tests for other medications.

#### Availability of Data

Another limitation is that some medications may not be tested by the Rouen University Hospital laboratories, meaning we either lack the data in our health data repository or the test does not exist at all. This lack of data could introduce bias by excluding certain medications from the evaluation process.

#### Multiple Appearances of a Single Patient

A single patient may undergo multiple genotyping or testing procedures during their hospitalization, resulting in multiple entries in the final section of the dashboard. This duplication can skew the data representation and will need to be addressed in future iterations of the dashboard.

Overall, while we aimed to minimize these biases and limitations, they are inherent to the current study design and data collection methods. Future work should focus on addressing these issues to improve the robustness and accuracy of the evaluation process.

To integrate the dashboard into clinical practice, we could start with pilot testing in select departments to gather feedback and make necessary refinements. The dashboard could then be integrated with hospital information systems to streamline workflows and ensure data privacy. Continuous monitoring and updates could be implemented to maintain the dashboard’s relevance and accuracy. For further research, we could focus on enhancing data standardization, expanding the scope of the dashboard to include more clinical data types, evaluating its long-term impact on patient outcomes, and exploring predictive analytics features to improve decision support and patient care.

## Conclusion

The dashboard for cytochrome-related DIs is population-based and aims to provide an overall view and raise awareness among prescribers about the importance of pharmacological dosages for their patients. Certain parameters can be improved and require additional work for each specific case. Ultimately, the tool can lead to an individualized approach and become a prescription assistance tool if integrated into a prescription support software.

## Supplementary material

10.2196/57705 Multimedia Appendix 1 Computer Usability Satisfaction Questionnaire.

## Abbreviations

CYP450: cytochrome P450
DI: drug interaction
DID: drug interaction dashboard
EDS: Entrepôt de Données de Santé
EDSaN: Entrepôt de Données de Santé Normand
FDA: US Food and Drug Administration
HDW: health care data warehouse
HeTOP: Health Terminology/Ontology Portal
HUG: University Hospitals of Geneva
LOINC: Logical Observation Identifiers Names and Codes
UCD: unit of common dispensing

## References

[1] Definition of cytochrome P450 enzyme system - NCI dictionary of cancer terms. National Cancer Institute.

[2] Guengerich FP (2021). A history of the roles of cytochrome P450 enzymes in the toxicity of drugs. Toxicol Res.

[3] (2002). Interactions médicamenteuses et cytochromes P450. PHARMA-FLASH.

[4] Ortiz de Montellano PR (2013). Cytochrome P450-activated prodrugs. Fut Med Chem.

[5] Jaladanki CK, Gahlawat A, Rathod G, Sandhu H, Jahan K, Bharatam PV (2020). Mechanistic studies on the drug metabolism and toxicity originating from cytochromes P450. Drug Metab Rev.

[6] Gu TM, Lewis JS, Le H, Bubalo JS (2022). Comparative effects of fluconazole, posaconazole, and isavuconazole upon tacrolimus and cyclosporine serum concentrations. J Oncol Pharm Pract.

[7] Bleyzac N, Bourguignon L, Goutelle S Tableaux de contribution CYP - DDI-predictor version académique. https://www.ddi-predictor.org/tools/cyp-contribution.

[8] Wishart DS, Feunang YD, Guo AC (2018). DrugBank 5.0: a major update to the DrugBank database for 2018. Nucleic Acids Res.

[9] Kato H (2020). Computational prediction of cytochrome P450 inhibition and induction. Drug Metab Pharmacokinet.

[10] Mishra NK (2011). Computational modeling of P450s for toxicity prediction. Expert Opin Drug Metab Toxicol.

[11] Simpao AF, Ahumada LM, Desai BR (2015). Optimization of drug-drug interaction alert rules in a pediatric hospital’s electronic health record system using a visual analytics dashboard. J Am Med Inform Assoc.

[12] Jeffries M, Gude WT, Keers RN (2020). Understanding the utilisation of a novel interactive electronic medication safety dashboard in general practice: a mixed methods study. BMC Med Inform Decis Mak.

[13] Satyam R, Yousef M, Qazi S, Bhat AM, Raza K (2021). COVIDium: a COVID-19 resource compendium. Database (Oxford).

[14] Dorsch MP, Chen CS, Allen AL (2023). Nationwide implementation of a population management dashboard for monitoring direct oral anticoagulants: insights from the Veterans Affairs Health System. Circ Cardiovasc Qual Outcomes.

[15] Teixeira V, Mori A, Usera A, Bacigalupo JC, Luna D (2019). Performance evaluation of clinical decision support systems (CDSS): developing a business intelligence (BI) dashboard. Stud Health Technol Inform.

[16] Iapadre G, Balagura G, Zagaroli L, Striano P, Verrotti A (2018). Pharmacokinetics and drug interaction of antiepileptic drugs in children and adolescents. Pediatr Drugs.

[17] Li ZR, Shen CH, Li RD (2023). Individual dose recommendations for drug interaction between tacrolimus and voriconazole in adult liver transplant recipients: a semiphysiologically based population pharmacokinetic modeling approach. Eur J Pharm Sci.

[18] Gosselin L, Letord C, Leguillon R (2023). Modeling and integrating interactions involving the CYP450 enzyme system in a multi-terminology server: contribution to information extraction from a clinical data warehouse. Int J Med Inform.

[19] Grosjean J, Merabti T, Dahamna B (2011). Health multi-terminology portal: a semantic added-value for patient safety. Stud Health Technol Inform.

[20] Pressat-Laffouilhère T, Balayé P, Dahamna B (2022). Evaluation of Doc’EDS: a French semantic search tool to query health documents from a clinical data warehouse. BMC Med Inform Decis Mak.

[21] HeTOP.

[22] Interactions médicamenteuses, cytochromes P450 et P-glycoprotéine (pgp). Hôpitaux Universitaires Genève.

[23] Interactions des médicaments. Centre Belge d’Information Pharmacothérapeutique.

[24] Drug Interactions Flockhart Table. Clinical Pharmacology - Indiana University School of Medicine.

[25] (2021). Drug development and drug interactions | table of substrates, inhibitors and inducers. US Food and Drug Administration.

[26] Comment mesurer l’UX et l’ergonomie de votre site web avec les echelles de mesure. Blog des Guiz.

