# Supplementary material for: Design and Implementation of a Dashboard for Drug Interactions Mediated by Cytochromes Using a Health Care Data Warehouse in a University Hospital Center: Development Study
Source: JMIR Med Inform. 2024 Nov 28;12:e57705. doi: 10.2196/57705 (PMC11620019; doi:10.2196/57705)
Supplement: Multimedia Appendix 1 [file medinform-v12-e57705-s001.docx]

**Multimedia Appendix 1**

Computer Usability Satisfaction Questionnaire.

| Computer Usability Satisfaction Questionnaire | | 1=Strongly disagree  2=Strongly agree  NA=not applicable | | | | | | | |
| --- | --- | --- | --- | --- | --- | --- | --- | --- | --- |
| **Utility of the tool** | | **1** | **2** | **3** | **4** | **5** | **6** | **7** | **NA** |
|  | In general, I find this tool easy to use |  |  |  |  |  |  |  |  |
|  | This tool is straightforward to use |  |  |  |  |  |  |  |  |
|  | I can perform my tasks efficiently using this tool |  |  |  |  |  |  |  |  |
|  | I can perform my tasks quickly using this tool |  |  |  |  |  |  |  |  |
|  | I can perform my tasks efficiently using this tool |  |  |  |  |  |  |  |  |
|  | I am comfortable using this tool |  |  |  |  |  |  |  |  |
|  | Learning to use this tool was easy |  |  |  |  |  |  |  |  |
|  | I believe I became productive quickly using this tool |  |  |  |  |  |  |  |  |
|  | I believe this tool is useful in my clinical practice |  |  |  |  |  |  |  |  |
|  | I believe this tool is useful in my epidemiology practice |  |  |  |  |  |  |  |  |
|  | I find this tool relevant to the practical reality |  |  |  |  |  |  |  |  |
| **Quality of information** | | **1** | **2** | **3** | **4** | **5** | **6** | **7** | **NA** |
|  | Error messages generated by the system clearly explain how to resolve issues |  |  |  |  |  |  |  |  |
|  | The information (on-screen messages) provided with the tool is clear |  |  |  |  |  |  |  |  |
|  | The information provided with the tool is easy to understand |  |  |  |  |  |  |  |  |
|  | The information is effective in helping me perform my tasks |  |  |  |  |  |  |  |  |
| **Ergonomics** | | **1** | **2** | **3** | **4** | **5** | **6** | **7** | **NA** |
|  | When I make a mistake using the tool, I can rectify them easily and quickly |  |  |  |  |  |  |  |  |
|  | The organization of information on the tool’s screens is clear |  |  |  |  |  |  |  |  |
|  | I need assistance in using this tool |  |  |  |  |  |  |  |  |
|  | I believe training is necessary to make the best use of this tool |  |  |  |  |  |  |  |  |
|  | It’s easy to find the information I need |  |  |  |  |  |  |  |  |
| **Interface quality** | | **1** | **2** | **3** | **4** | **5** | **6** | **7** | **NA** |
|  | The tool’s interface is pleasant |  |  |  |  |  |  |  |  |
|  | I enjoy using the tool’s interface |  |  |  |  |  |  |  |  |
|  | This tool has all the functionality and capabilities I expect |  |  |  |  |  |  |  |  |
| **Overall satisfaction** | | **1** | **2** | **3** | **4** | **5** | **6** | **7** | **NA** |
|  | Overall, I am satisfied with this tool |  |  |  |  |  |  |  |  |
| **Questions/comments** | |  | | | | | | | |
|  | Do you have any improvements in mind that the tool could have? |  | | | | | | | |
|  | Do you think this tool can be considered a means of communication to raise awareness about the need for pharmacological dosages? |  | | | | | | | |
|  | Any other remarks? |  | | | | | | | |
